# Proteogenomic analysis reveals Arp 2/3 complex as a common molecular mechanism in high risk pancreatic cysts and pancreatic cancer

**DOI:** 10.1038/s41598-025-87872-1

**Published:** 2025-01-31

**Authors:** A. K. M. Firoj Mahmud, Dina Gamaleldin Mansour Aly, Yelin Zhao, Mikael Benson, Martin Smelik, Oleg Sysoev, Hui Wang, Xinxiu Li

**Affiliations:** 1 Medical Digital Twin Research Group, Department of Clinical Science, Intervention and Technology (CLINTEC), Karolinska Institute, Stockholm, Sweden; 2 Division of Statistics and Machine Learning, Department of Computer and Information Science, Linköping University, Linköping, Sweden; 3 Jiangsu Key Laboratory of Immunity and Metabolism, Department of Pathogenic Biology and Immunology, Xuzhou Medical University, Xuzhou, 221000 Jiangsu China

**Keywords:** Predictive markers, Cancer genetics

## Abstract

Pancreatic cysts, particularly intraductal papillary mucinous neoplasms (IPMNs), pose a potential risk for progressing to pancreatic cancer (PC). This study investigates the genetic architecture of benign pancreatic cysts and its potential connection to PC using genome-wide association studies (GWAS). The discovery GWAS identified significant genetic variants associated with benign cysts, specifically the rs142409042 variant near the *OPCML* gene. A pairwise GWAS comparing PC to benign cysts revealed the rs7190458 variant near the *BCAR1* and *CTRB1* genes. Further analysis with identified GWAS genes highlighted the Actin Related Protein (Arp) 2/3 complex as a potentially important molecular mechanism connecting benign cysts and PC. The Arp2/3 complex-associated genes were significantly upregulated in PC, suggesting their role in the malignant transformation of pancreatic cysts. Differential expression of these genes was observed across various cell types in PC, indicating their involvement in the tumor microenvironment. These findings suggest that the Arp2/3 complex-associated genes can serve as potential biomarkers for predicting the malignant transformation of pancreatic cysts, opening new avenues for targeted therapies and early detection strategies.

**Supplementary Information:**

The online version contains supplementary material available at 10.1038/s41598-025-87872-1.

## Introduction

Pancreatic cysts are increasingly detected by different imaging methods. The majority of the cysts are benign, but a subset has the potential to progress to pancreatic cancer (PC)^1,2^.

PC is associated with a highly lethal malignant transformation and a poor prognosis. Thus, early diagnosis of pancreatic cysts that do or do not increase risk of malignancy is of great clinical importance. However, only about 0.25% of pancreatic cysts identified incidentally are cancerous, and less than 0.25% of these benign cysts transform into cancer within a year of their discovery ​^3^.

The transformation of pancreatic cysts from intraductal papillary mucinous neoplasms (IPMNs) to PC is often characterized by a sequence of dysplastic stages. It begins with low-grade dysplasia (LGD), advances to high-grade dysplasia (HGD), and invasive IPMN, which may ultimately lead to PC^4^. LGD represents an early, less severe form of pre-cancerous alteration, characterized by minimal cellular abnormalities. As the dysplasia progresses to HGD, the abnormalities become more pronounced, indicating a higher risk of transitioning to an invasive cancer. This progression highlights the critical need to monitor and understand the molecular and genetic architecture changes occurring within IPMNs. The presence of HGD is a strong predictor of the potential for malignancy and serves as an indicator for surgical intervention^4^.

Desipte advances in surgical thechniques, systemic treatment options for PC remain limited. Converntional chemotherapy and radiotherapy can only slightly prolong life expectancy by a few months^5^. Novel treatments, including targeted therapies, immunotherapy and optimized nutrution have been investigated to improve patient outcomes^6–11^.

However, there are many challenges to these treatmetns –including primary, adaptive, and acquired resistance; various immune-relate adverse events, and obstacles in transling research outcomes to determe the optimal personalized treatment^5,12–14^. These challenges and obstacles will require understanding of the mechanisms underlying transformation from LGD to HGD and further into invasive IPMN and PC. This could contribute to improved tools for early prediction and management of such transformations. Many studies have been conducted to find biomarkers for high-risk IPMN. However, to date, none have been incorporated into routine clinical practice due to their limited prognostic efficiency^15–18^. Another significant challenge in identifying biomarkers is the intralesional heterogeneity within IPMNs and PC. One potential solution is suggested by recent studies indicating that HGD IPMN may share molecular mechanisms with PC^19^. Genetic^20^ and cellular abnormalities observed in HGD IPMN show similarity to those seen in PC^21^. Thus, understanding of such abnormalities may contribute to identification early mechanisms underlying malignant tranformation of pancreatic cysts.

Genome-wide association studies (GWAS) have revolutionized the understanding of complex traits by identifying genetic variants associated with various diseases. GWAS could provide valuable insights into the genetic basis of various conditions and elucidate the genetic architecture to help in understanding disease mechanisms. Unfortunately, the genetic architecture of pancreatic cysts remains poorly understood. Previous studies have largely focused on genetic alterations in PC rather than on its potential precursors, namely pancreatic cysts. Genetic variants identified by GWAS can be used to find underlying disease mechanisms based on mapped genes. Such variants can also be used to construct polygenic risk scores (PRS) that indicate an individual’s risk for a specific disease, including malignant transformation^22–30^.

**Fig. 1:**
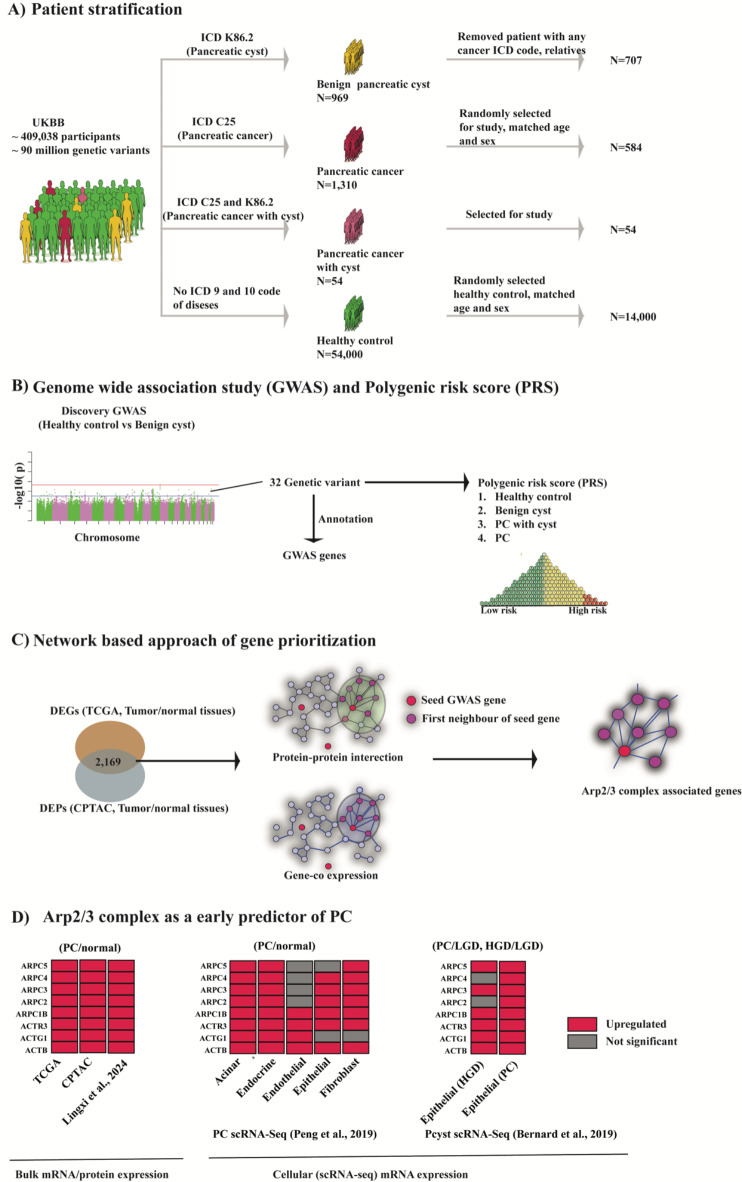
Overview of the study. (**A**) Genomic data from patients and healthy control were collected from UK Biobank (UKBB) and grouped based on and 9th and 10th revision of the International Statistical Classification of Diseases and Related Health Problems (ICD) 9 and 10 codes. (**B**) Discovery GWAS analysis was carried out between healthy control and benign cysts. PRSs were calculated for different groups. (**C**) GWAS gene and protein-protein interaction (PPI) along with gene co-expression revealed Arp2/3 complex associated genes as a link between genomic and functional regulation of pancreatic cyst and PC. (**D**) Arp2/3 associated genes/proteins were upregulated in PC patients at both bulk and cellular levels. In summary, we present the stepwise discovery of Arp2/3 complex by proteogenomic analysis and explore the regulation pattern of Arp2/3 complex associated genes/proteins at bulk and cellular levels to find its important role for PC progression from cyst to PC.

In our study, we aimed to define the genetic makeup of benign cysts and identify potential biomarkers in IPMN that share similar regulatory directions in PC at cellular levels. To achieve this, we performed discovery GWAS, and systematically evaluated the concordance of the changes in protein/mRNA expression in PC at bulk and single-cell levels (Fig. 1). GWAS-genes and commonly up and down-regulated proteins/genes in PC revealed that Actin Related Protein (Arp) 2/3 complex-associated genes/proteins were significantly upregulated in PC and high-risk pancreatic cysts.

## Results

### Genetic architecture of pancreatic cyst

**Fig. 2:**
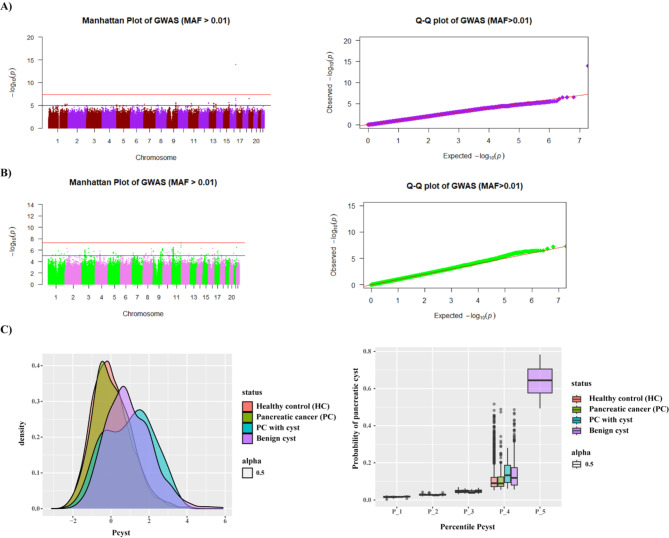
Manhattan and Quantile-Quantile (Q-Q) plots of GWAS of benign pancreatic cysts. (**A**) Discovery GWAS of 707 benign pancreatic cysts(benign cyst) cases versus 14,000 healthy controls. The rs142409042 near *OPCML* locus was associated with benign cysts (OR = 2.583 (1.835–3.636), *p* = 5.33 × 10^−08^). (**B**) Pairwise GWAS of 584 individuals diagnosed with PC (cases) versus 707 individuals with benign cysts (controls). The rs7190458 near *BCAR1* and *CTRB1* was associated with PC versus benign cysts (OR = 0.152 (0.094–0.244), *p* = 1.01 × 10^−14^). The red line on the Manhattan plots of (A) and (B) indicates the genome-wide significance threshold (*p* < 5 × 10^−08^) and the suggestive association is indicated by the blue line (*p* < 10^−05^). (**C**) PRS density plot and percentile plots, respectively. 65% of the individuals diagnosed with benign pancreatic cysts rather than PC were represented in the fifth percentile.

To our knowledge the genetic architecture of pancreatic cysts has not yet been characterized. The main goals of this study were to determine (1) the genetic architecture of benign pancreatic cysts using GWAS data (Fig. 1A, B) and (2) if this was associated with PC (Fig. 1C, D).

First, a discovery GWAS was performed to identify genetic associations in individuals with benign pancreatic cysts compared to healthy controls (707 individuals with benign pancreatic cysts (cases) and 14,000 healthy controls of European origin from the UK Biobank (UKBB). Additionally, a pairwise GWAS was performed to determine the genetic associations in individuals with PC compared to those with benign pancreatic cysts (584 individuals diagnosed with PC (cases) versus 707 individuals with benign pancreatic cysts (controls), supplementary Table S1). Individuals diagnosed with malignant cysts were excluded from both the discovery and pairwise GWAS analyses.

Analyses of the discovery GWAS identified rs142409042 genetic variant near the opioid binding protein/cell adhesion molecule like (***OPCML***) gene on chromosome 11 (Fig. 2A) has genome-wide significance association (*p* < 10^−08^) with benign pancreatic cysts versus healthy controls (odd ratio OR (95% confidence interval (CI) = 2.583 (1.835–3.636)). Similarly, the rs7190458 genetic variant near the BCAR1 scaffold protein, Cas family member (***BCAR1***) and chymotrypsinogen B1 (***CTRB1***) on chromosome 16 (Fig. 2B) showed a genome-wide significant association with PC versus benign pancreatic cysts (OR (95%CI) = 0.152 (0.094–0.244)). Notably, there was no overlap of genome-wide significant SNPs *p* < 10^−08^ between the discovery GWAS (benign pancreatic cysts versus healthy controls) and pairwise GWAS (PC versus benign pancreatic cysts) (Fig. 2C, supplementary Table S2, S3).

### Polygenic risk score (PRS) for prediction of the risk of developing benign pancreatic cyst

We next aimed to develop a PRS to predict the risk of benign pancreatic cysts. We refer to this PRS as benign pancreatic cyst PRS. Genetic variants identified in the discovery GWAS (benign pancreatic cyst versus healthy controls) were used to calculate PRS for each individual across the four groups: healthy controls, benign pancreatic cysts, PC, PC with cyst. To construct the PRS, 32 genetic variants from the discovery GWAS (*p* < 10^−05^) were weighted by their effect sizes. PRS calculation was performed for each individual, and percentiles were established based on the PRS of the healthy control group. Each individual from the four groups was assigned a percentile based on her or his PRS score. The logistic regression model and ANOVA were performed to compare benign pancreatic cysts, PC with cyst and PC with healthy controls. PRS showed a significant association with the risk to develop benign pancreatic cysts versus healthy controls (OR (95%CI) = 2.159 (2.017–2.311), supplementary Table S4). 65% of individuals with benign pancreatic cysts were represented in the upper percentile (Fig. 2C). In the lower percentile, the PC with cyst and PC were represented (ANOVA *p* < 2 × 10^−16^). A similar distribution pattern within the percentiles was noticed when analyzing females and males separately in supplementary Figure S1 and Table S4. This is consistent with the benign pancreatic cyst PRS being in a different range from PC and PC with cyst. Thus, benign pancreatic cyst PRS constructed from discovery GWAS were associated with benign cysts but not with PC, or PC with cysts (Fig. 2C).

However, since some pancreatic cysts become malignant, we next investigated whether the genes associated with benign cysts interacted with or overlapped with the genes or gene products associated with PC. For this purpose, we mapped the discovery and pairwise GWAS genetic variants to the corresponding genes. In the next section, discovery GWAS mapped genes are referred to as GWAS-genes. These GWAS-genes were used to test for overlap or molecular interactions with differentially expressed genes/proteins (DEGs/DEPs, respectively) from bulk and single cell transcriptomics or proteomics data. Interactions between GWAS-genes and DEGs/DEPs were sought based on the protein-protein interaction network from the STRING database.

### Arp2/3 complex as a common molecular mechanism between pancreatic cancer and pancreatic cyst

We next tested if there was any association between the GWAS-genes and mRNAs/proteins in PC. To define such mRNAs/proteins, we analyzed pancreatic tumor tissues and normal tissues from The Cancer Genome Atlas (TCGA) and Clinical Proteomic Tumor Analysis Consortium (CPTAC) to identify DEGs and DEPs, respectively (Tumor versus normal, adjusted p value (p-adjusted) < 0.05, absolute log fold change > 0.25). We identified 2,169 DEGs/DEPs that were consistently upregulated or downregulated in tumors across TCGA and CPTAC. Of these, six genes were the GWAS-genes, namely *NTM*,* ARPC3*,* CDCP1*,* FEZ2*,* FXYD2*, and *MYO1D*. All the corresponding mRNAs and proteins were upregulated in tumors, except *FXYD2* (supplementary Figure S2). We further hypothesized that the protein products of these six GWAS-genes interacted with these similarly upregulated or downregulated DEGs/DEPs within the tissue at a local level, potentially driving progression from pancreatic cysts to PC.

**Fig. 3:**
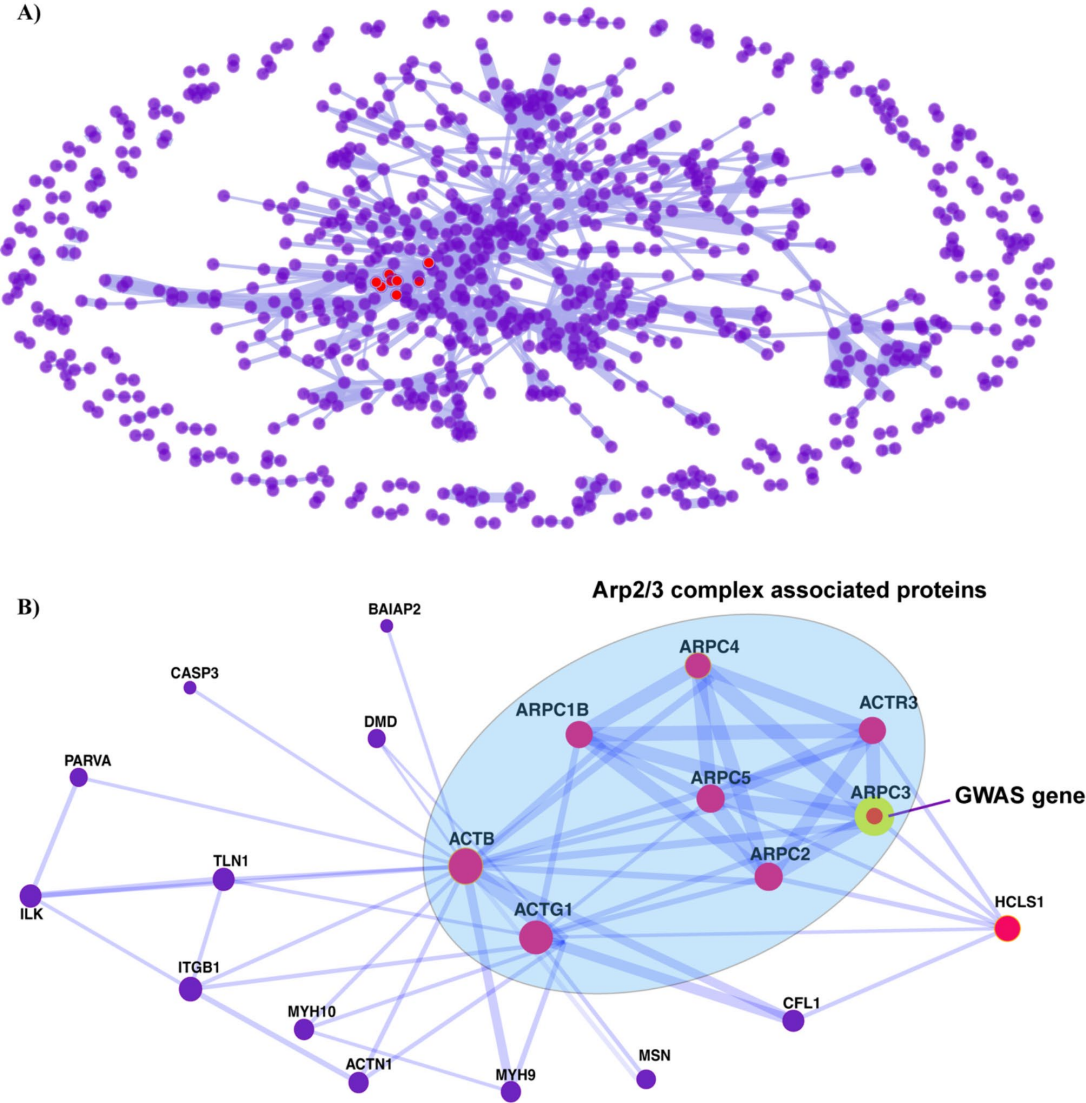
Protein-protein interactions (PPI) of concordant DEGs/DEPs in PC tumors from TCGA and CPTAC. (**A**) PPI network between 2,169 DEPs that showed similar regulatory directions as DEGs in PC. PPI (confidence score > 0.95) edge and thickness were based on co-expression values. GWAS genes and their first connected neighbors are highlighted in red. (**B**) GWAS gene *ARPC3* (yellow and red) and its neighboring genes (red) in the PPI network. Edge thickness indicates the degree of co-expression of highly connected genes in PC.

To test this hypothesis, we performed a PPI network analysis for the six GWAS-genes based on the STRING database. We utilized PPIs with a confidence score (> 0.95) to exclude spurious interactions random (a score of 1 represents the highest possible confidence in the STRING database). Additionally, we also considered co-expression of the proteins based on the STRING database to identify the co-expressed interaction partners. We found that only one GWAS-gene protein product, *ARPC3*, was highly connected in PPI network (confidence score > 0.95, Fig. 3A, supplementary Table S5). We further identified the primary neighbors of the ARPC3 protein, i.e. proteins that directly interacted with ARPC3, namely ARPC2, ARPC1B, ARPC4, ARPC5 and ACTR3 (confidence score > 0.95 based on PPI and co-expression, Fig. 3B) in PC. These DEPs were part of the Arp 2/3 complex. This complex, in turn, interacted with the effector downstream proteins including ACTG1 and ACTB, both of which were DEPs, with the highest possible confidence score (> 0.95) (Fig. 3B). The Arp2/3 complex genes mentioned above, as well as their downstream genes (henceforth referred to as Arp2/3 complex-associated genes), were linked with the actin-related pathways. These pathways included regulation of actin dynamics for phagocytic cup formation^31^.

To further examine the pathogenic relevance of ARP2/3 complex associated genes for PC we investigated the expression patterns of their mRNAs and proteins in bulk and single cell profiling data.

### Arp 2/3 complex-associated genes differed in gene expression and may be promising biomarkers in PC

**Fig. 4:**
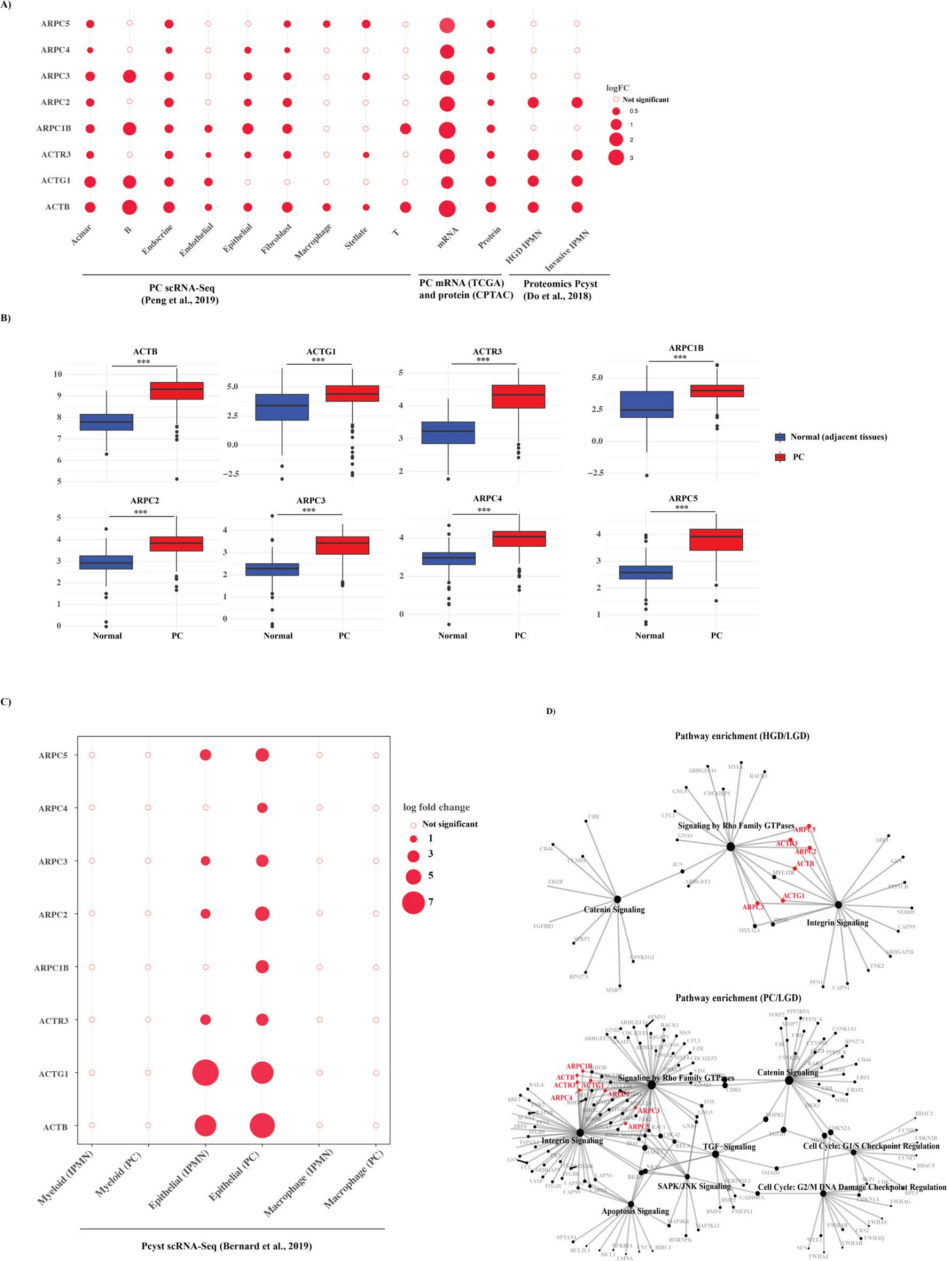
Expression of Arp2/3 complex-associated genes in PC and high-risk pancreatic cysts HGD IPMN. (**A**) Plots showing Arp2/3 complex-associated genes/proteins were differentially expressed in different cell types (scRNA-Seq), TCGA (mRNA), CPTAC (protein) and IPMN. (**B**) Plot showing that Arp2/3 complex-associated proteins were differentially regulated in PC compared to normal paired adjacent tissues (Lingxi et al., 2024). (**C**) Plot showing Arp2/3 associated gene expression changes in different cell types in HGD IPMN and PC compared to LGD IPMN. Six genes (*ARPC3*, *ACTB*, *ACTG1*, *ARPC2*, *ARPC5* and *ACTR3*) of the Arp2/3 complex were differentially expressed in HGD IPMN and PC epithelial cells compared to LGD IPMN. Circle size indicates fold change and red indicates upregulation. Circles without color showed changes that were not statistically significant. (**D**) Network graph showing pathways enriched for Arp2/3 associated genes, as well as the individual genes. Circle size corresponds to centrality. Arp2/3 complex associated genes which are differentially expressed are highlighted in red.

To investigate the regulation of Arp2/3 complex-associated genes/proteins in PC, we analyzed the DEGs and DEPs derived from comparing PC versus normal, based on data from TCGA and CPTAC. In summary, all eight genes and proteins associated with the Arp2/3 complex were significantly upregulated in PC patients in both TCGA and CPTAC datasets (absolute log fold change > 0.25 and p-adjusted < 0.05, Fig. 4A, supplementary Table S6). We first analyzed protein data from 191 PC tumor and 90 paired tumor-adjacent tissues^32^. The analysis showed that all of the eight proteins were significantly upregulated in the PC patients (absolute log fold change > 0.25, p-adjusted < 0.05) (Fig. 4A).

To further explore the potential relevance of Arp2/3 complex-associated proteins in premalignant lesions, we analyzed proteomics data from cyst fluid samples of nine IPMN patients^33^ classified into low-grade dysplasia (LGD, benign), high-grade dysplasia (HGD), and invasive IPMN. A comparison of LGD IPMN with HGD and invasive IPMN revealed 367 and 423 DEPs, respectively. Notably, among these DEPs, four Arp2/3 complex-associated proteins were consistently upregulated in both HGD and invasive IPMN (Fig. 4A).

We next extended our analysis to single-cell RNA sequencing (scRNA-seq) data to determine the presence of Arp2/3 complex-associated genes in specific cell types within the PC tumor microenvironment. We analyzed scRNA-seq data from 24 primary pancreatic tumors and 11 normal pancreases, encompassing 41,148 cells from tumor tissues and 15,444 cells from normal tissues to quality control and clustering. This process identified nine distinct cell types, each featuring 272 to 1,982 DEGs between tumor and normal tissues. Among these DEGs, genes *ACTB*, *ARPC2*, *ARPC3*, *ARPC4*, *ARPC5*, and *ACTG1* of the Arp2/3 complex-associated genes were notably upregulated across multiple cell types, including acinar, fibroblast, endocrine, and epithelial cells (Fig. 4B).

Taken together, these analyses supported that the Arp2/3 complex-associated genes may play an important role in the progression from pancreatic cysts to PC at cellular levels.

### Arp2/3 complex-associated genes as potential biomarkers for malignant transformation in pancreatic cysts

To systematically assess Arp2/3 complex-associated genes as potential biomarkers for malignant transformation from low-risk PC cysts to high-risk PC cysts, we explored whether a similar expression pattern of these genes was present in different cell types in HGD IPMN. We analyzed available scRNA-seq data from pancreatic cyst biopsies with HGD IPMN, LGD IPMN and primary pancreatic tumors^21^. After quality filtering, we analyzed 7,986 cells for cell typing and subsequent analyses. We identified two cell types: epithelial cells and macrophages (supplementary Figure S3). Due to low cell number/counts, we only focused on epithelial cells (*n* = 4,892 cells). Within this subset, we identified 327 DEGs for HGD IPMN (HGD versus LGD, absolute log fold change > 0.25, p-adjusted < 0.05) and 882 DEGs for PC (PC versus LGD) (supplementary Tables S7, S8). In epithelial cells, six genes associated with Arp2/3 were identified as DEGs in both PC and HGD IPMN when compared to LGD IPMN. These genes included *ARPC3*, *ACTB*, *ACTR3*, *ARPC2*, *ARPC5*, and *ACTG1*. In addition, *ARPC4* and *ARPC1B* were found to be differentially expressed in PC epithelial cells compared to LGD IPMN (Fig. 4C, supplementary Table S8).

Pathway enrichment analyses revealed that Arp2/3-associated genes, including *ARPC3*, *ACTB*, *ACTR3*, *ARPC2*, *ARPC5*, and *ACTG1*, were significantly enriched (p-adjusted < 0.05) in integrin signaling and signaling by Rho family GTPases in both epithelial cells of PC and HGD IPMN (Fig. 4D). These pathways, which are crucial for cellular adhesion and motility, may therefore be potentially relevant for malignant transformation in pancreatic cysts^34,35^.

## Discussion

Our study provided novel insights into the genetic architecture of benign pancreatic cysts and PC, which may contribute to identification of potential biomarkers for early detection and intervention. By using GWAS and subsequent proteogenomic analyses, we identified distinct genetic variants associated with benign pancreatic cysts and PC, pointing towards common molecular mechanisms involved in high-risk pancreatic cysts and PC.

In our discovery GWAS, we revealed a significant association between genetic variants near the *OPCML* in individuals diagnosed with benign cysts compared to healthy controls. *OPCML* gene is known to have tumor inhibitory effects in various cancers^36^, This suggests a potential genetic predisposition that protects the benign pancreatic cysts from developing to malignant by this tumor suppressor mechanism^36–39^. This finding highlights the potential of *OPCML* as a biomarker for identifying individuals with benign cysts that have lower probability to progress to malignancy.

Conversely, in our pairwise GWAS comparing PC patients to those with benign pancreatic cysts, we identified genetic variants near the well-known PC-associated gene *BCAR1*, on chromosome 16, which were significantly associated with PC compared to benign pancreatic cysts^40^. The lack of overlap in genome-wide significant SNPs between the discovery and pairwise GWAS suggests that benign pancreatic cysts and PC have distinct genetic architectures. This distinction is essential for understanding the molecular mechanisms underlying the malignant transformation in benign pancreatic cysts and may explain why not all cysts progress to PC.

To assess the predictive potential of these genetic findings, we developed a PRS based on 32 genetic variants from the discovery GWAS to predict the risk of developing benign pancreatic cysts. Our analysis demonstrated that 65% of individuals diagnosed with benign pancreatic cyst had higher PRS falling within the upper percentiles of the PRS distribution. In contrast, individuals diagnosed with PC did not present in the upper percentile as benign pancreatic cyst did. This suggests that our constructed PRS reflects a specific genetic signature for benign cysts, which can be used to distinguish benign cyst from PC. This finding indicated the potential of PRS as a predictive tool for benign pancreatic cysts and underscores the differences in genetic architecture underlying pancreatic cyst and PC.

To further explore the mechanisms underlying the transformation from benign pancreatic cyst to malignancy, we performed PPI network analysis using the genetic variants from our discovery GWAS (including genome-wide significant (*p* < 5 × 10^−08^) and the suggestive significance level (*p* < 10^−05^)) and differentially expressed proteins in PC. Our hypothesis was that PPI interactions between protein encoded by GWAS identified genes and differentially expressed proteins in PC could reflect mechanism underlying the transformation of benign pancreatic cysts to malignant PC.

Our PPI network analysis identified the Arp 2/3 complex as a key molecular mechanism linking benign cysts and PC. Specifically, *ARPC3*, identified in our GWAS data was significantly upregulated in PC. The corresponding ARPC3 protein of showed extensive interactions with other Arp2/3 complex proteins, suggesting its critical role in the malignant transformation of pancreatic cysts. The Arp2/3 complex is known to regulate cell motility and actin cytoskeleton, processes vital for cancer cell migration and invasion^41,42^. Further analysis revealed significant upregulation of Arp2/3 complex-associated genes in both bulk and scRNA-seq data from PC. The consistent upregulation across various cell types, including acinar, fibroblast, endocrine, and epithelial cells, supported an important role of the Arp2/3 complex in the tumor microenvironment. Previous studies have shown that silencing of the ARP2/3 complex subunits typically resulted in reduced cell migration capacity, and also, inhibition of the Arp2/3 complex affects tumor microtubule assembly and cellular connections in PC^42^. Zhao et al., demonstrated that the Arp2/3 complex influenced early pancreatic carcinogenesis by promoting cancer progression and alterations of actin dynamics^43^. Consistently, our pathway enrichment analysis revealed that Arp2/3-complex associated genes were linked to integrin signaling and Rho family GTPase activities, which are crucial for cellular adhesion and motility, thereby potentially influencing malignant transformation of pancreatic cysts and cancer progression.

These findings suggested that the Arp2/3 complex-associated genes or gene products could serve as potential biomarkers for the malignant transformation of pancreatic cysts and as targets for therapeutic interactions. The identification of the Arp2/3 complex-associated genes links back to our genetic architecture findings, providing a molecular mechanism that may explain certain genetic variants are related to cancer progression. Furthermore, proteomics data from cyst fluid samples bridges the gap between molecular findings and clinical applications. The observed upregulation of four Arp2/3 complex-associated proteins in HGD IPMN and invasive IPMN in the proteomics data sets^33^ highlights their relevance not only in malignant transformation but also in early detection in pancreatic cyst malignancy.

To access the clinical relevance of these findings, we performed survival analyses on PC patients from TCGA. These analyses revealed that PC patients with higher expression of the ARP2/3 complex-associated genes had significantly shorter overall survival than those with lower expression levels with a hazard ratio of 1.68 (95%CI, 1.04–2.72, *P* = 0.033) (supplementary Figure S4). This indicates that the ARP2/3 complex-associated genes not only plays a role in the malignant transformation of pancreatic cysts but also impacts patient prognosis, highlighting its potential target for therapeutic interventions and early detection strategies. Our study has several limitations. Firstly, genetic data for benign pancreatic cyst were only obtained from UKBB and there is no other genetic data from independent cohorts to replicate our GWAS findings. This may affect the generalizability of our results. Secondly, the analyses were based on limited bulk and single-cell omics data of pancreatic cysts. Additionally, functional validation of the identified genetic variants and their biological roles in pancreatic cyst progression was not directly performed in our study. Addressing these limitations will require future studies with larger and diverse cohorts as well as further experimental validation. Meanwhile, our study highlights several important areas for future research. Firstly, validation of our findings in larger cohorts is essential, along with an exploration of the functional roles of these genetic variants in the progression of pancreatic cysts to PC. Secondly, refining PRS models by incorporating additional genetic and environmental factors could enhance the accuracy of predicting benign pancreatic cysts and their potential transformation into malignancy. Investigating the intricate interactions between genetic variants and other molecular pathways involved in both pancreatic cysts and PC will provide a deeper understanding of their complex genetic landscape. Furthermore, developing targeted therapies that inhibit the Arp2/3 complex or modulate its activity may offer new avenues for treatment.

In conclusion, our study uncovers the distinct genetic architecture of benign pancreatic cysts and PC, identifies *OCPML* as a gene associated with benign cysts which is potentially protective against malignant transformation and links the Arp2/3 complex to the mechanisms underlying malignant transformation of pancreatic cysts. These findings may contribute to identify potential biomarkers and therapeutic targets for malignant pancreatic cysts, also offering avenues for the early detection and intervention. Looking to the future, we anticipate that further elucidation of molecular mechanisms underlying the malignant transformation from benign pancreatic cysts to PC will pave the way for the development of other novel diagnostic and therapeutic strategies. Over the next five or ten years, advancements in high-throughput single-cell and spatial single-cell RNA-sequencing, high-throughput microscopy and quantitative image with AI and advanced bioinformatics analyses are expected to enhance our understanding of the complex alterations in this process^44–49^. Integration of multi-omics data and collaborations across the different researchers or institutions from all over the world will accelerate the discovery of the reliable and feasible biomarkers and drug targets for malignant transformation.

Moreover, personalized medicine approaches are expected to become more prevalent. By incorporating genetic, environmental, and lifestyle factors to construct PRS models for individual patient could improve risk stratification and early detection. Targeted therapies designed to modulate the activity of specific genes or molecular complexes, such as the Arp2/3 complex identified in our study, may offer new treatment avenues.

## Methods

### UKBB participant stratifications and data analysis

Participants of this study were a part of the UKBB dataset, a large prospective cohort study consisting of more than 500,000 participants recruited in the United Kingdom^50^. Full details of the UKBB study can be found on the UKBB website (https://biobank.ndph.ox.ac.uk/showcase/). UKBB received ethical approval from the National Information Governance Board for Health and Social Care and the National Health Service, Northwest Multi-Center Research Ethics Committee^51^. All participants gave informed consent through electronic signatures before enrolment in the study. This research has been conducted under approved UKB Project ID 102,162. The data was downloaded, and thus the end of the follow up of the individuals was the 3rd of May 2023. The specific data fields used in this analysis were imputed genomic data, date of recruitment, age, sex, date of diagnosis, and Diagnostic codes ICD9 and ICD10.

The ICD10 codes was used to assess the diagnosis of the patients. We identified patient groups based on ICD10 codes in the hospital inpatient data (UKBB datasets field: 41270), which is curated from UKBB as provided. In the analysis, we included pancreatic cancer (ICD code C25 and Pancreatic cyst (ICD code K86.2). We only selected patients of European origin. For each group of patients, we identified a group of healthy controls, which were defined as all patients without any disease code ICD9 or ICD 10 codes. We used the MatchIt package^52^ to match the healthy control cases with the prevalent and incident cases based on age and sex. The match was performed by the nearest neighbor method using Euclidean distance as a measure of similarity. We used exact matching for sex. Patient characteristics are in supplementary Table S1.

We selected PC patients with cysts who have both ICD code C25 and K86.2 in the hospital inpatient data fields. Genetic data was downloaded from UKBB. The genotyping and imputation (and quality control) were performed by the UKBB^50^. Genome-wide data were available from the UKBB v3 imputed data in BGEN v1.2 format.

### Genome wide association analysis (GWAS)

Two case-control GWASs (Discovery GWAS and Pairwise GWAS) were performed using 90 million imputed autosomal genetic variants; single nucleotide polymorphisms (SNPs) from UKBB genetic data^50,53^. Details about the quality control and imputation of the genotyped genetic variants can be found in the UK Biobank_genotyping_QC_documentation^54^. All 707 individuals diagnosed with benign pancreatic cysts and 14,000 healthy controls were included in the discovery GWAS. 584 individuals diagnosed with (PC (cases) versus 707 individuals with pancreatic cysts (controls) were included in the pairwise GWAS to determine the differential genetic variants that are associated with the development of PC versus pancreatic cysts. Individuals diagnosed with pancreatic cysts and PC were excluded from both GWASs. SNPTEST v.2.5.2 was used to perform the GWAS analysis using the frequentist association analysis score method and adjusting for genetic principal components^55^. GWAS associations results were filtered based on minor allele frequency MAF (MAF > 0.01), Hardy–Weinberg equilibrium (*P* > 5 × 10^−7^) and imputation info scores (INFO > 0.4). Results visualization was done in R v.4.3.1 as Manhattan plots and Q–Q plots using the qqman and plyr packages^56^.

### Annotation of GWAS genes

National Center for Biotechnology Information (NCBI) SNP database, Variant Effector Predictor (VEP) and Functional mapping and annotation (FUMA version 1.5.2) were used to map the GWAS significant genetic variants (*p* < 10^−08^) and suggestive GWAS genetic variants (*p* < 10^−05^) to genes for both the discovery and pairwise GWAS. For the differential expressed genes and Protein-protein interaction section, we used only genes of the discovery GWAS which were referred to as GWAS-genes (supplementary Tables S2, S3, S9)^57,58^.

### Construction of polygenic risk score (PRS)

PRS were constructed from top genetic variants of the discovery GWAS (*p* < 10^−05^) as described in Mansour Aly et al.^59^. Genetic variants weighted by effect-size were used to calculate individual scores for four groups of individuals: benign pancreatic cyst, malignant pancreatic cyst, pancreatic cancer, and healthy individuals. Percentiles were calculated for PRS based on healthy controls; P_1 (< 20%), P_2 (20–40%), P_3 (40–60%), P_4 (60–80%) and P_5 (> 80%). Logistic regression models were performed for association analysis of the PRS using the scaled scores and adjusting for genetic principal components in R v4.3.1. All reported p values are two-sided.

### **Protein-protein interaction (**PPI**) network construction and subsetting**

PPI network was performed using STRING v.12.0^60^ database with the list of commonly up and downregulated DEGs/DEPs of TCGA and CPTAC. PPI and co-expression data were downloaded from the string data base on 12th February 2024. GWAS-genes and 2,169 common DEGs/DEPs were used to construct a PPI network. To ensure the highest probability of protein-protein interaction and minimize random interactions, we selected interactions with a confidence score greater than 0.95^60,61^. The reason for selecting the highest confidence score > 0.95 was to ensure the highest probability of PPI over randomly observing an interaction. Primary neighbors of GWAS genes were selected for the sub-setting of the network. Subset networks were selected if a GWAS gene was connected with any other protein in the network with a confidence score > 0.95 and by co-expression. The network generation, filtering, and figure generation were done by the R package Igraph 2.0.2.

### TCGA, CPTAC and proteomics data analysis

Gene count (STAR-count) matrices for 140 pancreatic tumor tissues and 76 normal pancreatic tissues (67 normal adjacent tissues and 9 normal ductal tissues) were downloaded by r package TCGAbiolink^62^ for differential expression analysis. Samples exhibiting low read counts or poor-quality metrics (50% of genes have zero counts in a sample) were excluded to ensure data integrity. Normalization of raw counts was performed using the variance-stabilizing transformation (VST) function from the DESeq2 (V 1.44.0) package in R by adjusting sequencing depth. Quality control checks, including principal component analysis (PCA) and hierarchical clustering, were conducted to identify and remove outliers and batch effects. Count data was modeled using a negative binomial distribution and differential expression was assessed by comparing tumor samples to normal samples. Genes with an p-adjusted (false discovery rate) < 0.05 and absolute log fold change > 0.25 were considered significantly differentially expressed (DEGs).

Proteomics data were obtained from the CPTAC, which included 140 tumor tissues and 76 normal tissues (67 normal adjacent tissues and 9 normal ductal tissues) of pancreas. Wilcoxon rank-sum test was used which is a non-parametric approach suitable for data without a normal distribution assumption to identify DEPs between tumor and normal tissues. DEPs were selected based on a threshold of fold change greater than 0.25 and an p-adjusted of less than 0.05, to account for multiple testing corrections and minimize false discovery rates. We used R version 4.1.0 for all the statistical analysis.

Protein abundance of three groups HGD IPMN, LGD IPMN and Invasive IPMN (three biological replicates × three technical replicates) was obtained from Do et al., 2017^33^. Proteins with at least six valid values within a histological group were selected for statistical analysis following the criterion established by the previous study^33^. This criterion was used to remove the nonspecific proteins to histological grades from the analysis. After the log2-transformation of protein intensities, the missing values were imputed using R package missForest which uses a random forest algorithm for imputation. Two two-way ANOVA test was then performed on the preprocessed dataset to detect differential expressed proteins (DEPs) between different histological IPMNs groups^63^. The comparative analysis was conducted for HGD versus LGD and Invasive IPMN versus LGD. Subsequently, Tukey HSD test was employed for the pairwise and an FDR threshold of 0.05 was applied to identify significant DEPs for each pairwise.

Normalized protein expression matrix of 191 PC tumor patients compared to 90 paired normal-adjacent tissues were downloaded from the study by Lingxi et al., 2024^32^. Wilcoxon rank-sum test was used to identify if Arp2/3 associated proteins were differentially expressed in PC tumor patients compared to paired tumor-adjacent tissues^64^. Statistical analyses were conducted using R version 4.1.0.

### scRNA-Seq data processing of PC tumors and control samples

We analyzed single-cell RNA-seq data from 24 pancreatic ductal adenocarcinoma (PC) tumors and 11 control pancreases, including samples from non-pancreatic tumor patients and non-malignant pancreatic tumor patients, obtained from GEO (GSA: CRA001160)^65^. The samples had similar gender distributions but differed in age (PC: mean 59.54 ± 8.67; controls: mean 47 ± 12.34; *p* = 0.008). Using Seurat v4.3.0, we pre-processed the data, filtering out cells based on mitochondrial content, read counts, and gene coverage. For quality control, cells with > 10% mitochondrial gene expression, < 200 detected genes were excluded. This resulted in a dataset comprising 41,148 cells from PC samples and 15,544 cells from control samples for further analysis^66^. We normalized each sample, identified 2000 variable features, and integrated the data using Seurat’s anchor-based methods^67^. The difference of gene expression between PC and normal cells was assessed using MAST with FindMarkers function, focusing on genes with an p-adjusted < 0.05 and an absolute log fold change > 0.25 to identify significant changes indicative of up- or downregulation in tumor tissues.

### scRNA-seq data processing of pancreatic cyst (HGD IPMN, LGD IPMN) and PC

We analyzed single-cell RNA-seq data from 2 high risk IPMN (HGD IPMN), 2 low risk IPMN (LGD IPMN) and 2 PC patients^21^. scRNA-seq count matrix were available on request^21^. Data preprocessing involved filtering cells based on mitochondrial content, read counts, and gene coverage, resulting in 17,847 cells from HGD IPMN, LGD IPMN and PC. For further analysis, we removed 1,344 cells that contained < 10% mitochondrial genes and < 200 genes. The dataset was processed in Python3 using Scanpy V2.0^68^ for numerical operations, including normalization, log-transformation, and scaling. Each sample was normalized, and batch effects were removed by ComBat command in Scanpy. Cell clusters and their marker genes were identified through PCA and clustering algorithms. For cell types, similar marker genes were used^21^ as well scRNA-seq of PC mentioned above. For differential gene expression analysis, MAST package^69^ in R, integrating an R connector rpy2 package within Python3 was used to facilitate the same DE analysis by MAST. DEGs were calculated between HGD IPMN vs. LGD IPMN and PC vs. LGD IPMN. Genes were marked as DEGs with a p-adjusted < 0.05 and an absolute log fold change > 0.25 to highlight significant up- or downregulation in HGD IPMN and PC compared to LGD IPMN.

To identify significant pathways of DEGs calculated by scRNA-seq data, we utilized the Molecular Signatures Database (MSigDB)^70^ R package. We selected relevant gene sets, including hallmark and curated gene sets (C2). The GSEA algorithm evaluated the enrichment of these gene sets in DEGs, providing enriching pathways and gene list in the pathways. The adjusted p-values were calculated using the Benjamini-Hochberg (BH) method. We selected significant pathways if p-adjusted value was < 0.05.

## Supplementary Information

Below is the link to the electronic supplementary material.

Supplementary Material 1

Supplementary Material 2

## Data Availability

Raw data from the UKBB is available for approved researchers through the UKBB data-access protocol. STAR-count TCGA data is available in GDC data portal (https://portal.gdc.cancer.gov/projects/TCGA-PAAD). CPTAC data were downloaded from (https://proteomic.datacommons.cancer.gov/pdc/). PC scRNA-seq data were available under Accession number GSA: CRA001160. scRNA-seq data were available on request 10. Any processed data (except UKBB) are available on request to mahmud.firoj@imbim.uu.se.
