# Supplementary material for: Proteogenomic analysis reveals Arp 2/3 complex as a common molecular mechanism in high risk pancreatic cysts and pancreatic cancer
Source: Sci Rep. 2025 Jan 31;15:3902. doi: 10.1038/s41598-025-87872-1 (PMC11785783; doi:10.1038/s41598-025-87872-1)
Supplement: Supplementary file 1 — Supplementary Material 1 [file 41598_2025_87872_MOESM1_ESM.docx]

**Supplemetary Figures**


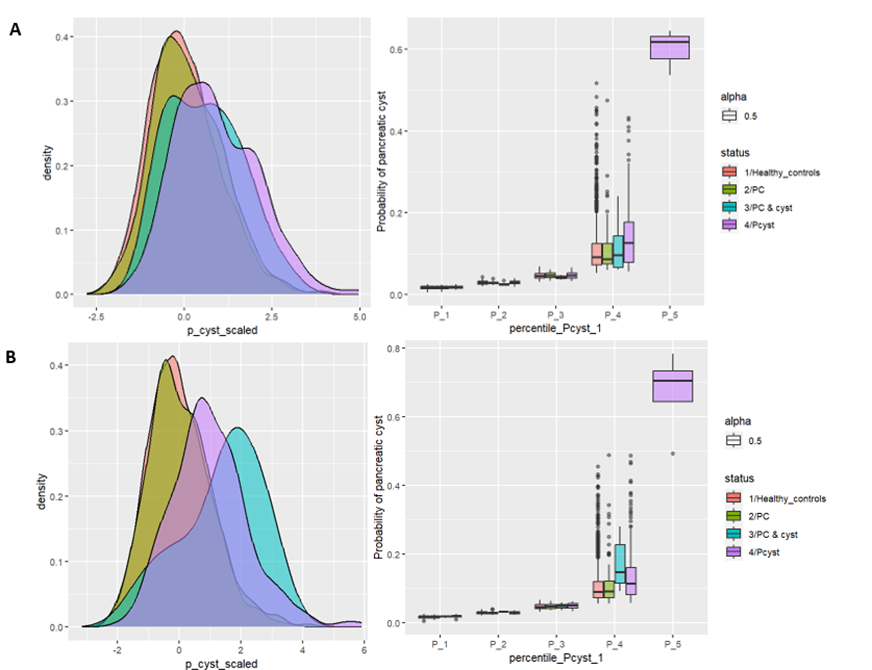


**Figure S1.** Genetic risk of benign pancreatic cyst estimated by benign pancreatic cyst PRS. The representation of pancreatic cyst (Benign cyst), malignant pancreatic cyst (malignant cyst) and pancreatic cancer (cancer) within the percentiles of polygenic risk score (PRS) in females and males, separately. **A).** represents the density plot (left) and the probability (right) of developing pancreatic cyst in females (nHC=7438, nPC=271, nPC&cyst=18, nPcyst=403) **B).** represents the density plot (left) and the probability (right) of developing pancreatic cyst in males (nHC= 5178, nPC=313, nPC&cyst=17, nPcyst=304).


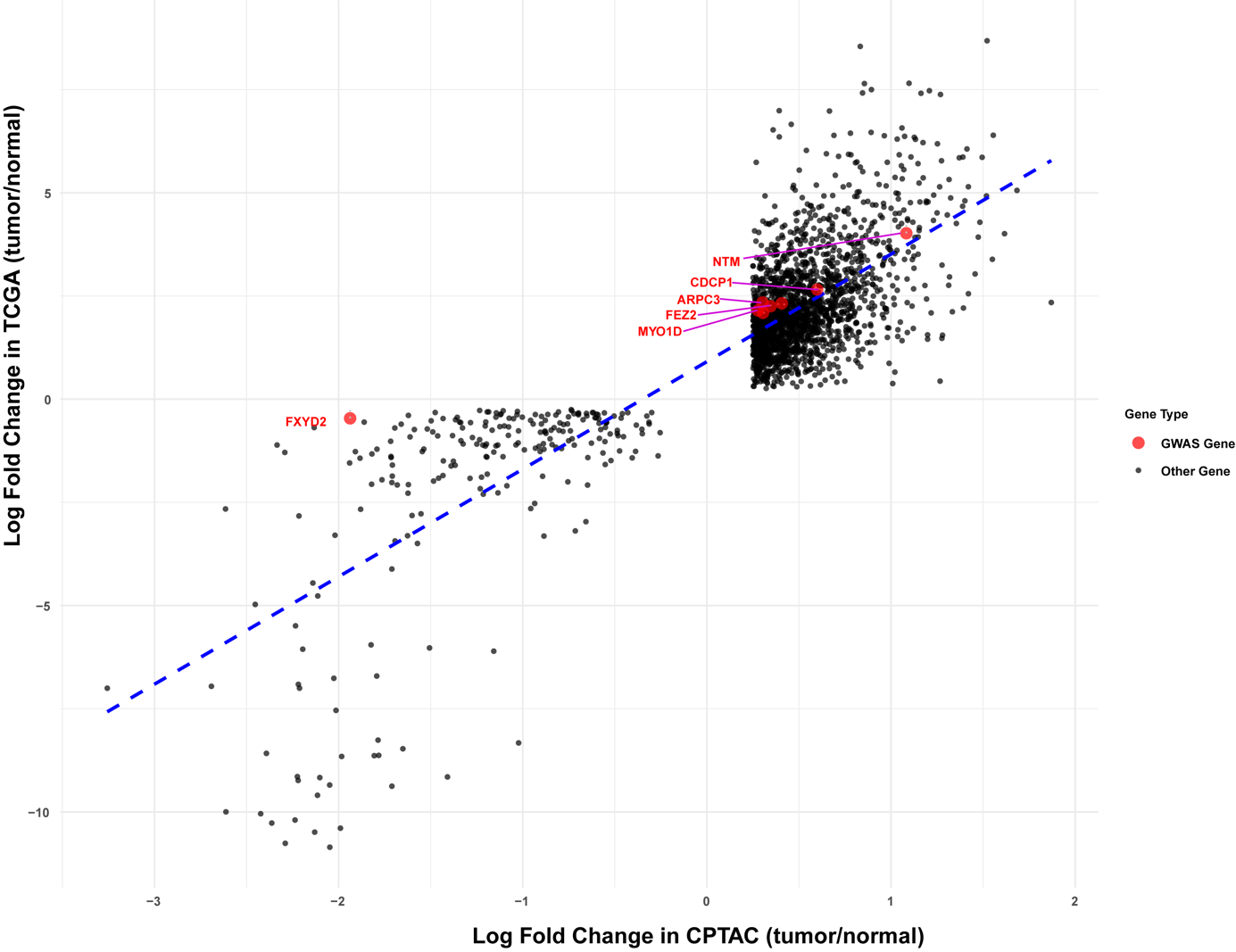


**Figure S2**: Scatter plot showing differentially expressed proteins (DEPs) of PDAC tumor in the X axis and differentially expressed genes (DEGs) in the Y axis. Highlighted genes in red indicated the GWAS genes that separated cyst from PDAC.

**
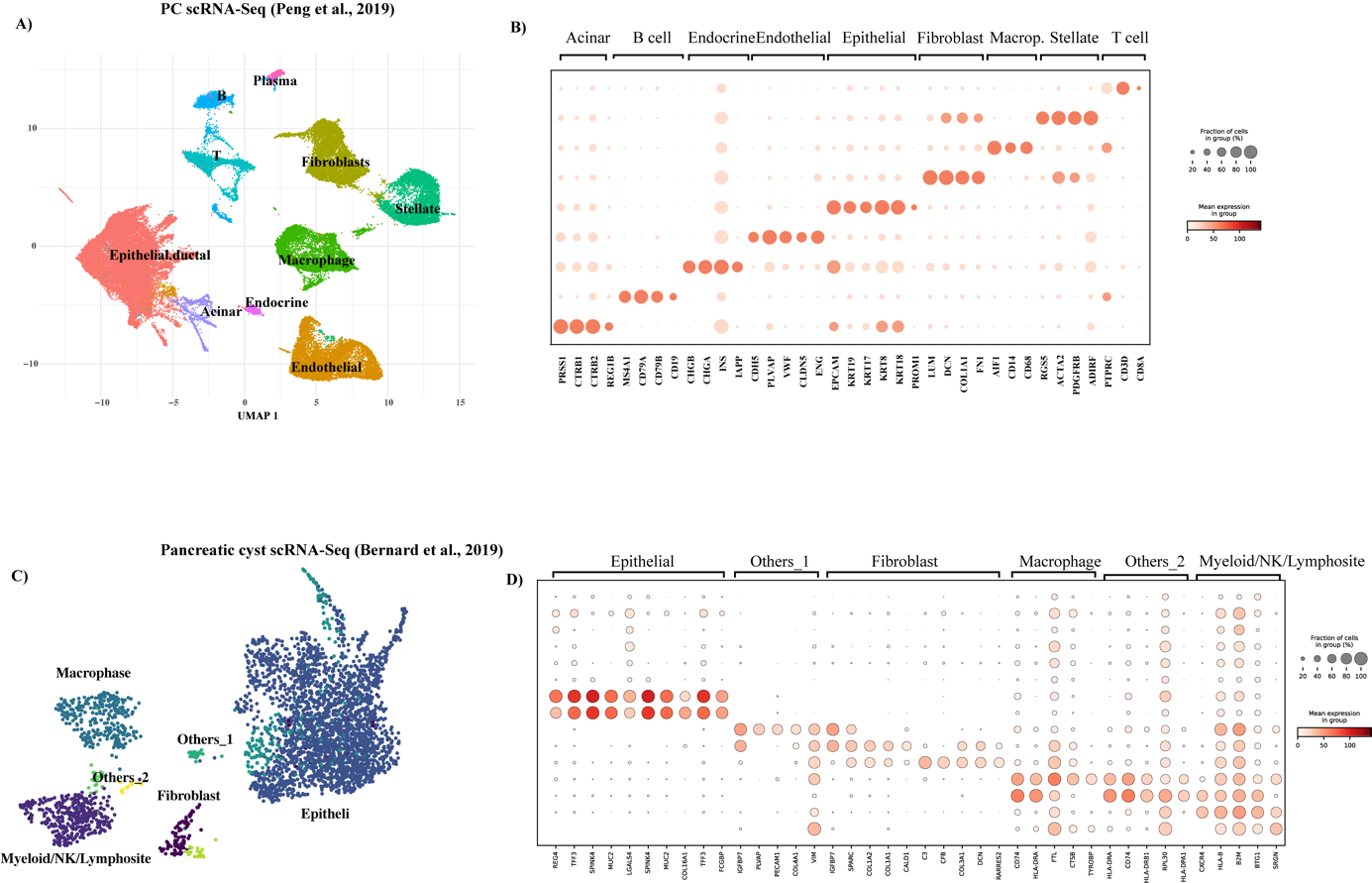
**

**Figure S3: scRNA-seq data of PC and pancreatic cyst. A).** UMAP plot showing cell clustering of PC scRNA-seq. Clustering of single cells from pancreatic cancer, highlighting distinct cell populations. **B).** Cell type annotation of PC scRNA-seq. Identification and labeling of cell types within the pancreatic cancer clusters. **C).** UMAP plot showing cell clustering of pancreatic cyst scRNA-seq **D).** Cell type annotation of pancreatic cyst scRNA-seq. Identification and labeling of cell types within the pancreatic cyst clusters. Others were mentioned when the cells were not distinctly grouped into any cell types.

**
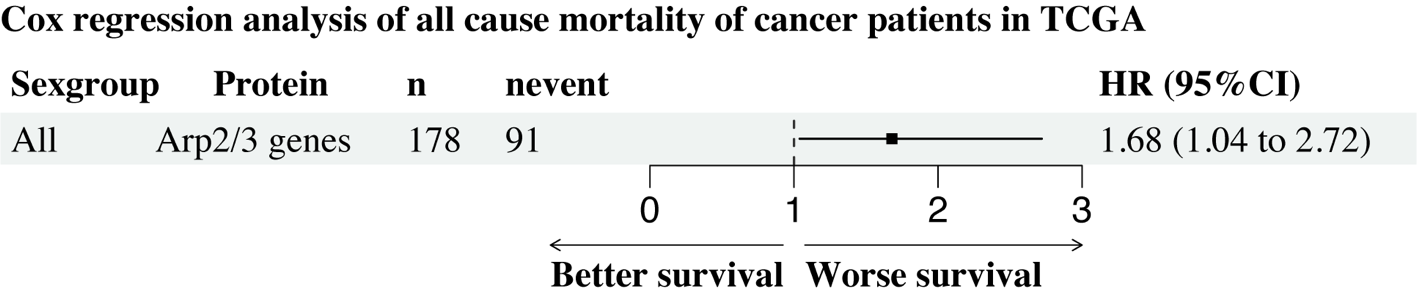
**

**Figure S4:** Survival plot of 178 PDAC patients from TCGA data based on 8 genes of Arp2/3 complex. n is the total number of cases.
